# Reconstruction of the Oral Cavity Using Facial Vessel-Based Flaps—A Narrative Review

**DOI:** 10.3390/cancers17172890

**Published:** 2025-09-02

**Authors:** Emilia Lis, Weronika Michalik, Jakub Bargiel, Krzysztof Gąsiorowski, Tomasz Marecik, Paweł Szczurowski, Grażyna Wyszyńska-Pawelec, Andrzej Dubrowski, Michał Gontarz

**Affiliations:** 1 Students’ Scientific Group of the Department of Cranio-Maxillofacial Surgery, Jagiellonian University Medical College, 30-688 Cracow, Poland; emilia.lis@student.uj.edu.pl (E.L.); weronika.michalik@student.uj.edu.pl (W.M.); 2 Department of Anatomy, Jagiellonian University Medical College, 31-034 Cracow, Poland; andrzej.dubrowski@uj.edu.pl; 3 Department of Cranio-Maxillofacial Surgery, Jagiellonian University Medical College, 30-688 Cracow, Poland; jakub.bargiel@uj.edu.pl (J.B.); krzysztof.gasiorowski@uj.edu.pl (K.G.); tomasz.marecik@uj.edu.pl (T.M.); pawel.szczurowski@uj.edu.pl (P.S.); grazyna.wyszynska-pawelec@uj.edu.pl (G.W.-P.)

**Keywords:** oral cavity reconstruction, local flaps, facial artery, FAMM flap, nasolabial flap, submental flap

## Abstract

Background: Reconstruction of oral cavity defects following oncologic surgery presents both functional and esthetic challenges. While microvascular free flaps remain the gold standard for large defects, local flaps based on facial vessels offer effective solutions for small-to-medium-sized reconstructions. This narrative review evaluates the clinical utility of three commonly used facial vessel-based flaps: facial artery musculomucosal, submental, and nasolabial flaps. Methods: A literature search was conducted using PubMed, targeting studies from 1981 to 2025 that described the anatomical basis, harvesting techniques, modifications, indications, advantages, disadvantages, complications, limitations, and outcomes of these flaps. A total of 73 full-text articles were included based on predefined inclusion criteria. Results: The FAMM flap provides versatile intraoral coverage with low donor site morbidity. The submental flap is effective in select patients, particularly those unfit for microvascular surgery, though its oncologic safety in neck-positive cases is limited. The nasolabial flap offers good esthetic and functional results with minimal external scarring. Each flap presents unique attributes regarding reach, vascularity, and complication profiles. Conclusions: Facial vessel-based flaps are viable and effective options for oral cavity reconstruction in appropriate clinical scenarios. Thorough knowledge of flap anatomy, indications, and oncologic considerations is essential for optimal outcomes.

## Simple Summary

Reconstruction of the oral cavity following cancer resection is essential for restoring function and appearance. While free flaps are commonly used for larger defects, they are not applicable for all patients due to surgical complexity or health conditions. This review focuses on three types of local flaps that are based on facial vessels—facial artery musculomucosal, submental, and nasolabial flaps. These flaps offer reliable alternatives for small-to-medium-sized defects with shorter operation times, fewer complications, and lower costs. Each flap has its own strengths and limitations depending on the defect location and patient characteristics. By providing detailed anatomical and clinical insights into each flap type, this review aims to support head and neck surgeons in choosing the most effective reconstruction strategy for various clinical cases.

## 1. Introduction

Reconstruction of oral cavity defects following cancer resection presents a unique and complex challenge for head and neck surgeons, where the restoration of both esthetics and function is essential. The anatomy of the oral region not only demands precise surgical technique but also underscores the evolving complexity of achieving optimal functional and esthetic outcomes. A wide range of reconstructive strategies are available, depending on the complexity and size of the defect, ranging from simple excisions or skin grafts to more complex locoregional pedicled flaps and free microsurgical flaps [1,2]. Among these options, pedicled flaps based on the facial or buccal vessels have proven particularly valuable for the successful reconstruction of small-to-medium-sized head and neck defects, offering wide arcs of rotation, reliable blood supply, and minimal donor site morbidity [3,4]. Over the past few decades, the use of local facial vessel-based flaps, such as the submental flap, the nasolabial flap, and the facial artery musculomucosal (FAMM) flap, has increased, further demonstrating their versatility in intraoral reconstruction [5,6,7]. Nonetheless, microvascular free flaps remain the recommended approach for the reconstruction of more extensive or complex head and neck defects [2,4].

**Figure 1:**
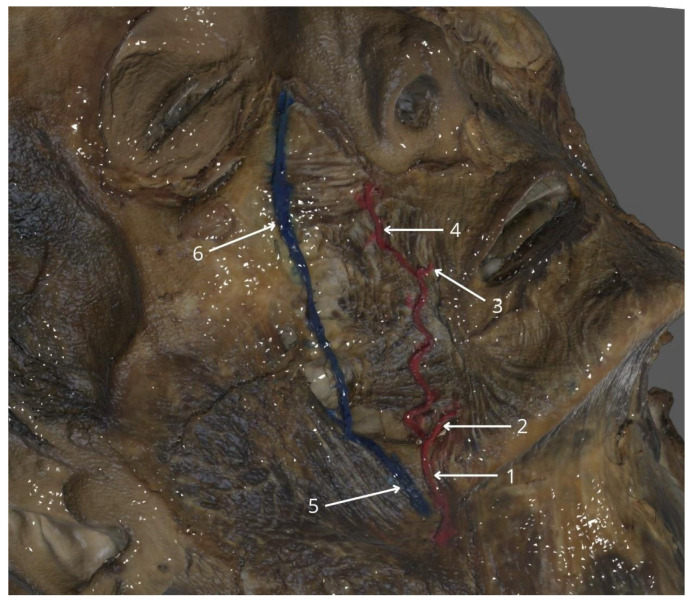
Facial vessels with their branches on a three-dimensional (3D) scan of a human cadaveric specimen, presenting (1) right facial artery, (2) right inferior labial artery, (3) right superior labial artery, (4) right angular artery, (5) right facial vein, and (6) right angular vein. This 3D scan of a cadaveric specimen was prepared at the Department of Anatomy, Jagiellonian University Medical College, in Kraków, Poland, and has not been previously published.

The facial artery (FA) is the main vascular structure in facial surgery, particularly for locoregional flap techniques in oral cavity reconstruction (Figure 1). Awareness of common variations, including facial and cervical branches, is important in minimizing intraoperative complications and optimizing flap design [8,9]. Originating from the external carotid artery, the FA follows a path across the neck and face, providing blood supply via its major branches: the submental artery, inferior labial artery, superior labial artery, lateral nasal artery, and angular artery, as a terminating branch [10,11]. Its predictable proximity to anatomical landmarks such as the mandibular angle, oral commissure, and nasolabial fold makes it an ideal pedicle for intraoral flaps like FAMM. The primary trunk of the FA usually runs in close proximity to the nasolabial fold on the face. Yang et al. reported that although anatomical position varies, it can be noted within 5 mm of the nasolabial fold in over 40% of cases, crossing the fold in one-third of cases. The artery courses between superficial and deep facial muscles, providing both vascular reliability and surgical accessibility. A comprehensive understanding of the topography of the branches of this artery is essential for ensuring the safety of flap harvesting and achieving optimal outcomes in reconstructive procedures of the oral and perioral regions [8]. The FA demonstrates a progressive reduction in diameter as it courses from its origin to its terminal branches. In the submandibular region near its origin from the external carotid artery, the FA typically measures approximately 2.5–3.0 mm in diameter. As it ascends over the mandibular border and enters the facial region, its external diameter decreases to a mean value of 1.9 mm at the level of the mandible body and around the nasolabial fold, it narrows further to approximately 0.8 mm [12]. This tapering pattern reflects the vessel’s branching and diminishing perfusion requirements along its distal path.

The facial venous system comprises a dense and interconnected network essential to the superficial and deep venous drainage of the face. The facial vein, a principal superficial vessel of the face, begins as the angular vein at the medial canthus and courses obliquely across the face to drain into the internal jugular vein. It receives multiple tributaries, including the buccal vein, which drains the buccinator muscle, buccal fat pad, and adjacent facial structures. The buccal vein typically joins the facial vein near the anterior border of the masseter, although anatomical variability is common, with communications noted between the buccal, deep facial, and transverse facial veins [13]. These venous connections form a valveless system that allows for bidirectional flow. Clinically, this is highly significant, particularly in procedures involving the midface, perioral region, and pterygomaxillary space. The presence of anastomoses between the facial vein and deeper venous systems, such as the pterygoid venous plexus via the deep facial vein, provides a route for retrograde spread of infection from the “danger triangle” of the face to the cavernous sinus, with potential for life-threatening complications such as cavernous sinus thrombosis [14]. This knowledge is crucial to avoid uncontrolled bleeding incidents, especially during facial flap elevation, buccal fat pad harvest, or tumor resections in the cheek and infratemporal fossa. In contrast, a valveless system offers the potential to harvest flaps with retrograde vein flow. Furthermore, the veins’ proximity to branches of the facial nerve demands meticulous dissection to prevent iatrogenic nerve injury. The facial artery and vein are located at variable distances from each other which can be most profoundly noted around the oral commissure and the nasal base [15]. Intraoperative awareness of these pathways seems vital for successful surgical outcomes in maxillofacial reconstruction procedures.

This narrative review discusses the potential applications of facial vessel-based flaps in the surgical reconstruction of defects in the oral cavity following cancer resection. Furthermore, a comprehensive evaluation of the anatomical structure, harvesting techniques, indications, contraindications, advantages, and disadvantages of each flap was conducted. The primary objective of this review is to enable the optimal selection of post-resection reconstructive techniques, thereby enhancing the healing of both donor and recipient sites, and thus improving patients’ capacity to obtain the most advantageous outcomes.

## 2. Materials and Methods

### 2.1. Search Strategy

The literature search was conducted using the PubMed database to gather all relevant studies on facial vessel-based flaps. The following search terms were used: ((oral cavity) OR (oral cavity reconstruction) OR (oral and maxillofacial reconstruction) OR (pedicled flap) OR (facial artery) OR (local flap) OR (facial artery musculomucosal flap) OR (FAMM flap) OR (myomucosal flap) OR (submental flap) OR (nasolabial flap) OR (nasolabial island flap)). Neither the date, article type, or text availability conditions were applied. An additional search was conducted through the references of the identified studies at the end of the search stage to ensure the precision of the process.

### 2.2. Eligibility Assessment

**Figure 2:**
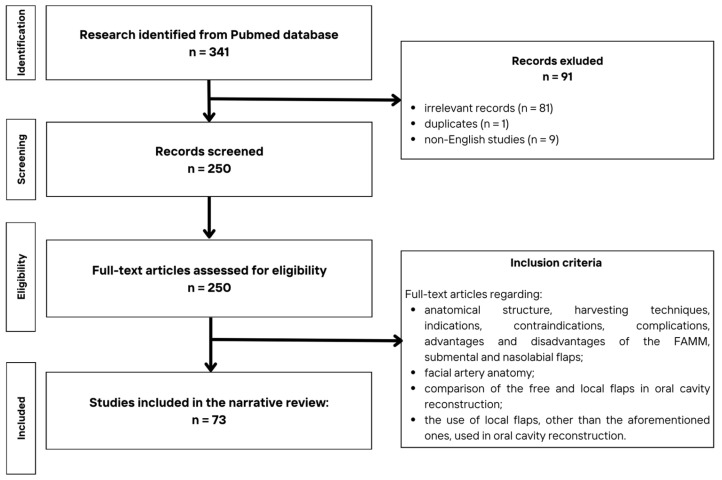
The flow diagram of the search procedure.

The flow diagram of the search procedure is provided in Figure 2. Initially, after the search of the database and an additional manual search through the references, a total of 341 studies were identified and first reviewed by two independent reviewers (E.L. and W.M.). Duplicates, irrelevant records, and non-English studies were excluded from further analysis. A total of 250 studies were qualified for full-text evaluation. The inclusion criteria involved full-text articles regarding anatomical structure, harvesting techniques, indications, contraindications, complications, advantages and disadvantages of FAMM, submental, and nasolabial flaps; facial artery anatomy; comparison of free and local flaps reconstruction of the oral cavity; and the use of other local flaps, other than the aforementioned ones, in oral cavity reconstruction. Finally, a total of 73 studies were included in this narrative review.

This narrative review was conducted and assessed in accordance with the SANRA (Scale for the Assessment of Narrative Review Articles) checklist to ensure methodological transparency and quality.

## 3. Results

### 3.1. Facial Artery Musculomucosal Flap (FAMM)

The facial artery musculomucosal flap was first described by Pribaz et al. in 1992 as a universal solution for intraoral and oropharyngeal soft tissue reconstruction [16]. Designed to address small-to-medium-sized defects, the FAMM flap gained popularity due to its ease of harvest, reliable vascular supply, and favorable tissue characteristics that closely match the oral mucosa [17].

#### 3.1.1. Flap Anatomy

Anatomically, the FAMM flap is an axial composite flap based on the FA in the buccal area and includes the FA, the buccal mucosa, the submucosa, the buccinator muscle, and the superficial layer of the orbicularis oris muscle in the area of the oral commissure [16]. The flap can be designed as either superiorly pedicled (based on retrograde flow via the angular artery) or inferiorly pedicled (based on antegrade flow from the FA) [17,18]. The facial vein is usually not included in the flap as venous drainage is assured by a submucosal plexus. The flap is typically up to 7 cm long, with a maximum width of approximately 2.5 to 3 cm. It can be made longer if it is extended further up into the gingivolabial sulcus [17,18,19].

#### 3.1.2. Flap Indications

The versatility of pedicle orientation allows the FAMM flap to reconstruct defects in various intraoral locations, including the buccal mucosa, floor of the mouth, tongue, palate, alveolar ridge, inner surface of the lips, and vermillion [3,4]. It is also occasionally used for the closure of oroantral and oronasal fistulae, as well as for providing well-vascularized tissue in cases of post-radiation necrosis [20,21]. A newly explored application is skull base reconstruction, as demonstrated in two studies utilizing the superiorly based FAMM flap [22,23].

#### 3.1.3. Flap Harvesting

The FAMM flap is designed with careful anatomical considerations to ensure optimal functionality and vascularity. In the inferiorly based FAMM flap, the outline is usually marked on the buccal mucosa, with the anterior limit placed 1 cm posterior to the oral commissure to avoid its distortion and the posterior boundary defined by the orifice of Stensen’s duct. The initial step in harvesting the FAMM flap is identifying the FA, either distally or anteriorly. Although a Doppler may be used to confirm the artery’s location, it is usually unnecessary, as the vessel typically lies within predictable anatomical landmarks [3,17,24]. Distal identification involves making an incision through the mucosa, submucosa, and buccinator muscle until the FA is located. Alternatively, the anterior approach begins approximately 1 cm posterior to the oral commissure to identify the superior labial artery, which is then traced retrogradely to the main trunk of the FA. Flap elevation involves dissection deep in the FA, incorporating part of the buccinator muscle along the flap’s length and including the orbicularis oris muscle near the commissure.

The FA should remain attached to the overlying tissues throughout the dissection [3,17,18,24]. The flap pedicle width, measuring 2 to 3 cm, is centered over the second and third lower molar regions. The flap is oriented obliquely, following the trajectory of the FA from the second molar to the ipsilateral gingivolabial sulcus. Venous drainage of the flap is primarily via the submucosal plexus, so inclusion of the facial vein is not necessary. However, preservation of a soft tissue base approximately 2 cm in width is essential to ensure adequate venous outflow [17,18].

**Figure 3:**
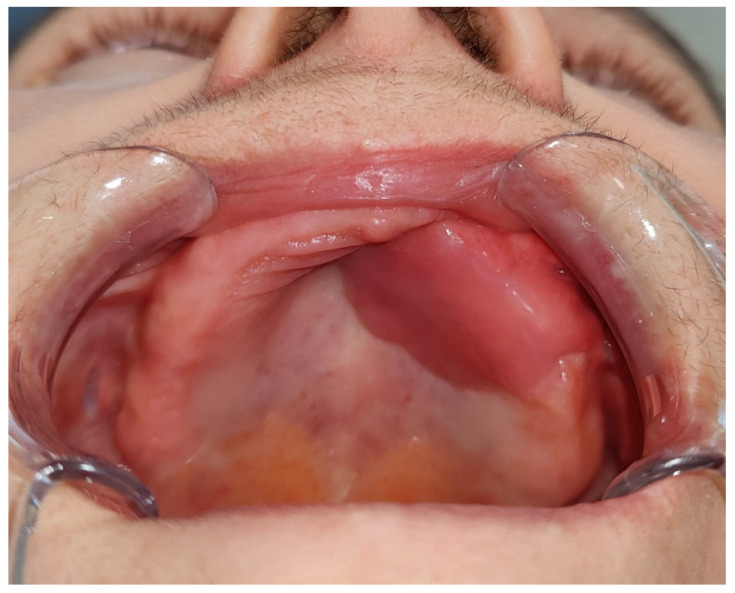
Clinical view of a 65-year-old female patient following partial resection of the left maxilla due to squamous cell carcinoma of the upper gingiva, with reconstruction using a superiorly based FAMM flap, performed at the Department of Cranio-Maxillofacial Surgery, Jagiellonian University Medical College, Kraków, Poland.

For a superiorly based FAMM flap, the base is located in the superior gingivolabial sulcus near the alar margin, with the distal end extending to the retromolar trigone. This type of flap can be pedicled on the angular artery via retrograde flow from the external carotid artery system. If the superior labial artery represents the terminal branch of the FA, it may serve as the flap’s pedicle, provided that retrograde flow is sufficient [7,25] (Figure 3).

The donor site can be closed primarily if the flap is less than 3 cm wide. In cases where closure under tension is a concern, alternatives include skin grafting, secondary healing by granulation, or closure using a buccal fat pad flap. If needed, the pedicle may be safely sectioned approximately three weeks postoperatively [17].

#### 3.1.4. Flap Modifications

**Figure 4:**
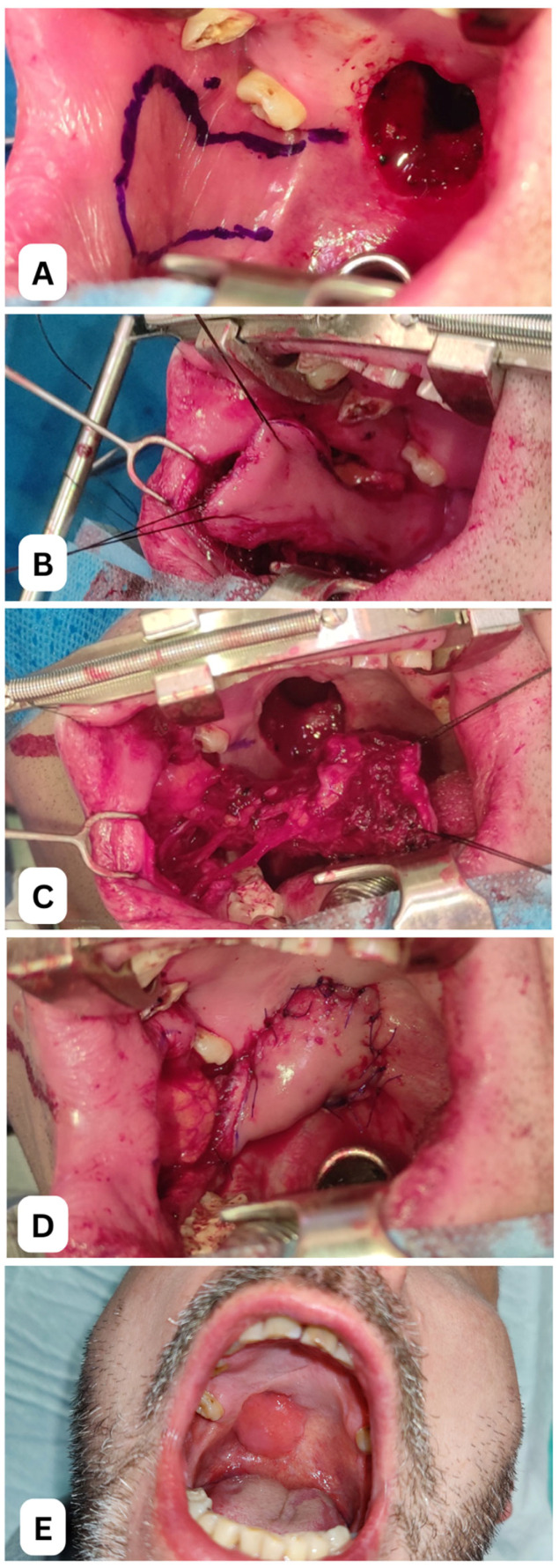
Clinical case of a 38-year-old male patient following excision of a low-grade mucoepidermoid carcinoma of the right hard palate with associated bone resection. (**A**) Intraoperative view of the palatal defect with marked dpFAMM flap and visible Stensen’s duct orifice. (**B**) External view during harvesting of the dpFAMM flap. (**C**) Ventral view of the FAMM flap with clearly visible facial vessels. (**D**) Reconstruction of the palatal defect using the dpFAMM flap; the donor site was reconstructed with a buccal fat pad. (**E**) Late outcome two years postoperative, following remodeling of the dpFAMM pedicle, showing proper mouth opening. All images depict a patient treated at the Department of Cranio-Maxillofacial Surgery, Jagiellonian University Medical College, Kraków, Poland.

The FAMM flap can be modified in several ways to improve its suitability for intraoral reconstruction. One such modification is the double-pedicled FAMM flap (dpFAMM), which incorporates both the facial and buccal vessels. In this design, the facial vessels form the lateral pedicle, while the buccal vessels constitute the medial pedicle. The dpFAMM flap typically measures 6–7 cm in length, with an anterior width of 4–5 cm and a posterior width of approximately 3 cm at the mucosal pedicle. If greater width is required, the central portion of the flap can be expanded by dividing and transposing Stensen’s duct [26]. The primary technical challenge in harvesting a dpFAMM flap lies in preserving the integrity of both the facial and buccal vascular pedicles. When successfully executed, this modification offers enhanced vascularization, improving flap viability. The presence of additional venous drainage through the buccal veins and submucosal plexuses also makes the dpFAMM flap more resistant to venous congestion and necrosis, even in patients who develop postoperative neck hematomas or who had undergone previous neck dissection [27] (Figure 4).

**Figure 5:**
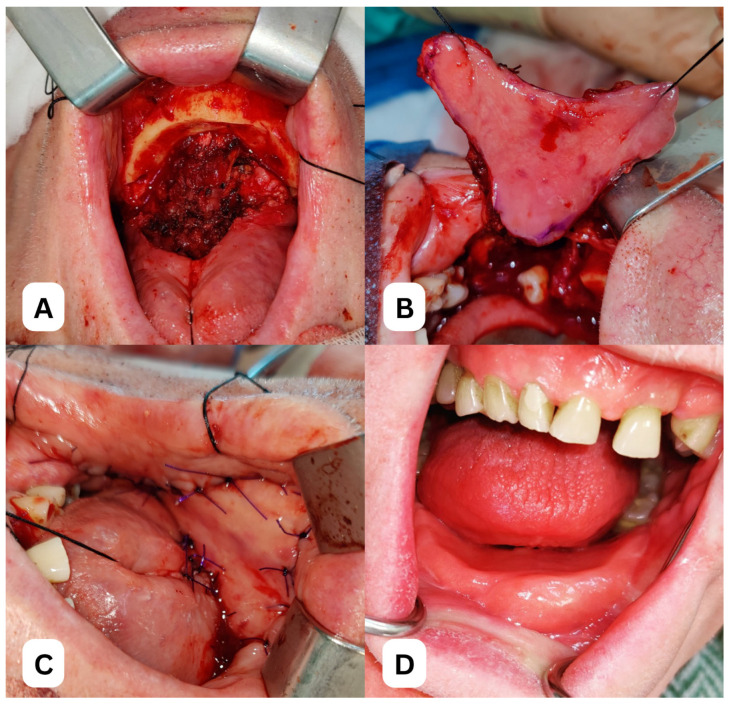
Clinical case of a 78-year-old male patient following marginal resection of the mandible due to squamous cell carcinoma of the lower gingiva. (**A**) Intraoperative view of the defect. (**B**) Harvesting of the iFAMM flap. (**C**) Reconstruction of the lower gingiva and floor of the mouth using the iFAMM flap. (**D**) Late outcome 18 months after surgery and postoperative radiotherapy. All images depict a patient treated at the Department of Cranio-Maxillofacial Surgery, Jagiellonian University Medical College, Kraków, Poland.

The island FAMM (iFAMM) flap is a further flap modification. In iFAMM flaps, the pedicle is isolated from the surrounding tissue, allowing for greater flexibility in the flap’s design and movement. By fully dissecting the facial vessels and creating a tunnel between the cheek and mandible—followed by another tunnel connecting the neck to the oral cavity—the flap can be repositioned independently of the teeth, which can avoid the need for secondary procedures to section the pedicle [24,28]. As the flap is tunneled lingually under the mandibular body, it is especially useful in patients with full dentition. This flap is valuable in repairing defects in the palate, pharynx, tongue, and floor of the mouth. Despite its advantages, the island FAMM flap is technically more demanding and requires a longer operative time. The primary risk associated with this technique is an increased likelihood of flap necrosis, most often due to kinking or compression of the vascular pedicle during tunneling [17,24,28,29] (Figure 5).

The free FAMM flap involves completely harvesting the flap from its donor site with arterio-venous pedicles and transferring it to a distant area for microsurgical reconstruction [29]. The free FAMM flap is particularly advantageous for reconstructing complex or anatomically distant defects, where local or regional options are inadequate. However, despite being a potential option for reconstruction using a FAMM flap, this method is not widely used. The literature contains only a single case report describing the application of this technique [17,29].

#### 3.1.5. Flap Advantages and Disadvantages

Among the FAMM flap benefits are the relative ease of harvesting, reliable and predictable vascularity, low donor site morbidity, no external scarring, and reduced operative time compared to free flaps [3,4]. Moreover, FAMM flaps provide excellent color and texture match as well as an immediate mucosal lining, making them highly suitable for intraoral soft tissue reconstruction [17,28]. 

Nonetheless, several limitations must be carefully considered. The most significant disadvantage is the limited size of the flap. Contraindications include the absence or compromise of the FA, as the flap’s viability is dependent on a preserved vascular supply from this vessel. Furthermore, patients with a previous neck dissection with facial vessel ligation and radiotherapy may exhibit insufficient vascularity, which can adversely affect flap survival [17]. In such cases, the use of a dpFAMM flap with preserved buccal artery blood supply could be a potential alternative [27]. Furthermore, oncologic safety is a critical consideration. A thorough evaluation of the neck’s vascular status is essential before planning reconstruction with a flap based on the facial vessels. The presence of metastasis in lymph nodes can complicate dissection of the facial vessels, making the procedure either technically challenging or oncologically unsafe [30]. The summary of advantages and disadvantages of various FAMM flaps is visualized in Table 1.

**Table 1:** Comparison of the advantages and disadvantages of different types of FAMM flaps.

Flap Type	Advantages	Disadvantages
**FAMM flaps in general**	No external scar—excellent esthetic outcome Perfect color match and mucosal surface with no hair growth Thin and pliable Easy to harvest Multiple modifications described Flap harvest and tumor ablation performed in the same operative field (one team) Economically feasible Low donor site morbidity Low complication rate	Limited width and size Requires use of a bite block postoperatively to prevent pedicle injury in dentate patients Bulky flap may hinder use of dental prosthesis when used in vestibular reconstruction Requires rehabilitation of oral cavity function May cause intraoral scarring, tethering, or reduced cheek mobility Not suitable if the FA is absent, damaged, or sacrificed in prior surgery May require second surgery section and modeling of the pedicle
**Modifications**		
**Double-pedicled FAMM**	Includes both facial and buccal vessels, improving flap vascularity and increasing arc of rotation and versatility Lower risk of flap necrosis and venous congestion Can be used for reconstruction of larger defects if designed with anterior widening Can be used after facial vessel ligation and prior radiotherapy Flap harvest and tumor ablation performed in the same operative field (one team)	Requires a careful dissection around two vascular pedicles Limited size and width Sometimes use of a bite block or tooth extraction in dentate patients is necessary May cause intraoral scarring, tethering, or reduced cheek mobility May require second surgery section and modeling of the pedicle
**Island FAMM**	Larger arc of rotation—can reach more distant intraoral areas Single-stage procedure More compatible for dentate patients	Technically demanding and more time-consuming Higher risk of venous congestion—any tension or kinking in the pedicle can reduce perfusion May cause intraoral scarring, tethering, or reduced cheek mobility
**Free FAMM**	Maximum reach and versatility—can be used to reconstruct distant defects within or outside the oral cavity Allows tailored inset and revascularization Useful when local flaps or vessels have already been used or compromised	Requires microsurgical expertise and longer operating time Higher risk of flap failure—as with all free flaps, success depends on reliable microvascular anastomosis

#### 3.1.6. Complications

Several complications are commonly associated with the use of FAMM flaps, as reported in the literature. These include partial or complete flap necrosis, dehiscence, venous congestion, hematoma, and infections. Two factors that significantly increase the risk of partial flap necrosis are previous neck dissection and prior radiation therapy [7,17]. Nonetheless, a meta-analysis by Mattey et al. reported a pooled success rate of 99.47% for the use of FAMM flaps, with a pooled overall complication rate of 30.18%, underscoring the low failure and complication rates [31].

#### 3.1.7. Functional Outcomes

The functional outcomes of oral cavity reconstruction using the FAMM flap are generally satisfactory, despite the fact that reconstruction of the tongue and other oral cavity structures presents a significant challenge. A study by Navarro Cuéllar et al. revealed that 86.3% of patients achieved normal oral opening, with a high incidence of excellent lingual tip elevation, protrusion, and lateral excursion [32]. Additionally, Jowett et al. reported complete preservation of oral competence, maintenance of tongue mobility, and excellent word articulation following FAMM flap reconstruction for floor-of-mouth defects [18]. However, reconstruction of complex structures, such as the tongue, remains a significant challenge due to its role in speech, swallowing, and mastication. Achieving optimal functional recovery requires a comprehensive rehabilitation plan, which focuses on restoring speech articulation and swallowing function. Speech therapy plays a crucial role, particularly for patients with floor-of-mouth or tongue defects, where muscle retraining and adaptation are necessary for normal function.

### 3.2. Submental Flap

#### 3.2.1. Flap Anatomy

The submental flap, first described by Martin et al. in 1993, is a regional flap based on the submental artery, cervical branch of the FA deep in the submandibular gland [5]. The flap typically includes skin, subcutaneous fat, and platysma, and may incorporate the anterior belly of the digastric muscle, enhancing its vascular reliability. The submental artery provides one to four perforators, ensuring consistent perfusion, making the flap highly dependable even when designed as a myocutaneous or osteomyocutaneous variant [33]. Its vascular pedicle length ranges from 5 to 8 cm, offering adequate reach to reconstruct intraoral, oropharyngeal, and lower facial defects [34].

#### 3.2.2. Flap Indications

The primary indications for the submental flap include reconstruction of small-to-medium-sized defects of the oral cavity (floor-of-mouth, buccal mucosa, tongue), lower face, and oropharynx, particularly in oncologic patients, where free flap options may not be feasible due to patient comorbidities or lack of microsurgical expertise [35]. The flap has also been successfully used in post-traumatic, post-infectious, and congenital deformities, showcasing its versatility. It is particularly advantageous in elderly patients and those with compromised vascular status, where microsurgical reconstruction carries higher risk of free flap necrosis [36].

#### 3.2.3. Flap Harvesting

**Figure 6:**
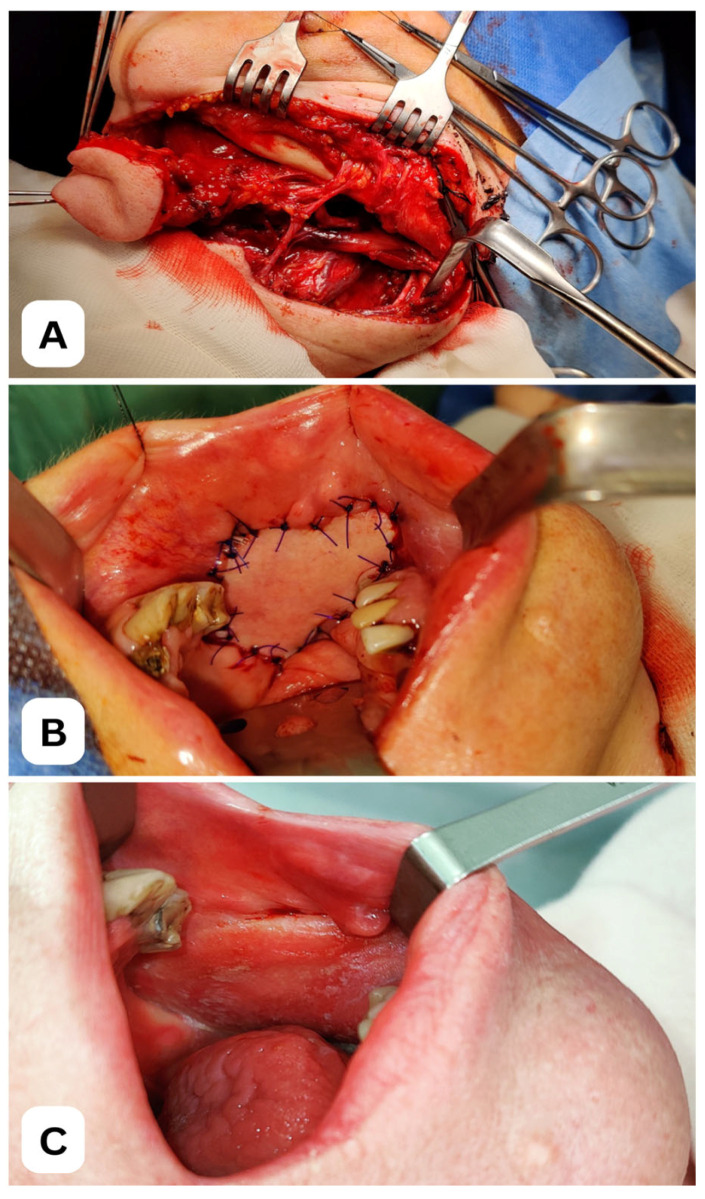
Clinical case of a 52-year-old female patient following selective, elective neck dissection (levels III–I) due to squamous cell carcinoma of the left buccal mucosa. (**A**) Intraoperative view of the harvested submental flap (3 × 5 cm) with preserved facial vessels. (**B**) Reconstruction of the left buccal mucosa using the submental flap. (**C**) Late outcome 26 months after surgery and postoperative radiotherapy. All images depict a patient treated at the Department of Cranio-Maxillofacial Surgery, Jagiellonian University Medical College, Kraków, Poland.

First, the elliptical shape for the skin island is drawn, with the upper edge of the incision lying just below the mandibular margin from angle to angle. To determine primary closure, the lower limit and width of the flap are assessed through a skin pinch test [37]. Harvesting the submental flap begins with an incision approximately 1.5 cm below the inferior border of the mandible, extending from the midline to the mandibular angle. The flap is elevated in a subplatysmal plane, and the submental artery is identified deep in the anterior belly of the digastric muscle [38]. Careful dissection is required to preserve the marginal mandibular branch of the facial nerve, and skeletonization of the pedicle may be performed to maximize reach [39]. When additional bulk is needed, the anterior belly of the digastric and the mylohyoid muscle may be included. Reverse-flow and perforator-based modifications have been developed to expand the arc of rotation and minimize donor site morbidity [33,40] (Figure 6).

#### 3.2.4. Flap Advantages and Disadvantages

Several advantages provide the popularity of the submental flap: proximity to the defect allowing a single-stage procedure, and a well-hidden donor site scar within the submental crease [41]. Moreover, it does not require microvascular anastomosis, reducing operative time and technical demands. Its thin, pliable tissue is well suited for intraoral lining, and when muscle is included, it provides sufficient bulk for three-dimensional reconstruction [42]. However, disadvantages must be considered. The flap’s reach is limited mainly to defects above the mandibular border, and its use is contraindicated in patients with clinically positive level I cervical lymph nodes due to the risk of oncologic compromise [38,43]. Furthermore, concerns exist regarding potential injury to the marginal mandibular nerve, and in cases where the vascular anatomy is aberrant or compromised, flap viability may be at risk [34]. For esthetic and functional outcomes, the hair-bearing nature of the submental flap can be problematic in individuals with very hairy necks, particularly for intraoral reconstruction. This issue is managed with techniques such as laser hair removal, secondary procedures, mechanical depilation, or electrolysis. Also, hair growth is limited after postoperative radiotherapy.

#### 3.2.5. Complications

Complications reported with the submental flap include venous congestion, partial/total flap necrosis, infection, and donor site complications such as hematoma or dehiscence [34]. Marginal mandibular nerve paresis, though typically temporary, is a notable risk, ranging from 0% to 17% [44]. Despite these potential issues, the flap demonstrates high survival rates in the literature, often exceeding 90%, with low rates of major complications [37,41].

#### 3.2.6. Functional Outcomes

Functional outcomes following submental flap reconstruction have been typically documented as excellent. Patients typically regain satisfactory speech, swallowing, and oral competence, with minimal impact on mandibular contour or function when a careful technique is employed [37]. Esthetic outcomes are also remarkably fine, with donor site scars well concealed under the chin and minimal long-term morbidity. Patient satisfaction is high, particularly when balanced against the simplicity and reliability of the flap compared to free flap alternatives [45].

### 3.3. Nasolabial Fold Flap

#### 3.3.1. Flap Anatomy

The nasolabial flap, first described by Esser in 1918, remains a time-tested and reliable option for maxillofacial reconstruction, particularly suited for defects of the midface and intraoral regions. It is based on the rich vascular supply of the angular artery, a terminal branch of the FA, and can be designed as superiorly or inferiorly based depending on the defect’s location [46]. The flap consists of skin, subcutaneous tissue, and sometimes underlying muscle from the nasolabial fold, a natural skin crease that allows well-camouflaged donor site scars [47].

#### 3.3.2. Flap Indications

Indications primarily include reconstruction of small-to-medium-sized defects of the oral cavity (especially the floor of the mouth, tongue, buccal mucosa, and gingiva), nasal sidewall, and lower eyelid, often following oncologic resection or facial trauma [48]. The flap is especially useful in elderly patients or those cases when prolonged surgery or microsurgical free flap reconstruction are contraindicated [49].

#### 3.3.3. Flap Harvesting

Harvesting the nasolabial flap involves marking the flap along the nasolabial crease, elevating it in a subcutaneous or submuscular plane, depending on the design, and preserving the vascular pedicle carefully [50]. Depending on the cheek laxity and size of the recipient defect, typically 4 to 5 cm of flap is harvested. A superiorly based flap can reach the nasal and periorbital regions, while an inferiorly based flap is typically used for intraoral reconstruction, with the flap passing through a subcutaneous tunnel to reach intraoral defects [46].

#### 3.3.4. Flap Modifications

Modifications include the island flap variant for greater arc of rotation, the bilateral nasolabial flap for larger defects, and incorporation of muscle or cartilage for additional support [51].

#### 3.3.5. Flap Advantages and Disadvantages

Advantages of the nasolabial flap include its simplicity of harvest, proximity to recipient sites, and excellent color and texture match, especially in midfacial reconstructions [52]. Additionally, donor site morbidity is minimal, and the scar usually blends well with the natural crease. The previous literature reported almost 100% survival rate of this flap type in oral cavity reconstruction [51,53]. However, its disadvantages include limited flap size, which restricts its use to relatively small defects, and the risk of trapdoor deformity or bulkiness, particularly when used intraorally [54]. In cases of prior surgery or radiation in the cheek region, vascular reliability may be compromised [55].

#### 3.3.6. Complications

Complications associated with the nasolabial flap are generally minor but can include distal tip necrosis, hematoma, infection, and hypertrophic scarring at the donor site [52]. The width of the flap is determined by the laxity of the tissues in the nasolabial region so it is a more useful flap in elderly patients. To reduce the tension during donor site closure, subcutaneous plane undermining in the cheek and M-plasty are commonly suggested techniques [56]. Venous congestion, particularly in island flap variants, and wound dehiscence are also reported but are typically manageable with conservative measures [49]. The risk of orocutaneous fistula and iatrogenic dermoid cyst should also be considered.

#### 3.3.7. Functional Outcomes

Functional outcomes following nasolabial flap reconstruction are correlated with good restoration of oral competence, speech, and swallowing function in intraoral cases [53]. Esthetic outcomes are highly favorable, especially in nasal and midfacial reconstructions, owing to the excellent skin match and concealed donor site. Patient satisfaction is consistently high, making the nasolabial flap a valuable reconstructive option in appropriately selected patients [54,57].

## 4. Discussion

Reconstruction of defects in the oral cavity represents one of the most complex and challenging procedures in the field of surgical practice. The challenge lies not only in the location of the defects in the facial region, but also in the complexity of restoring functions related to speech and swallowing. The outcomes of various reconstructive methods depend on the surgical technique, the extent of the procedure, and individual patient characteristics. Local flaps are an effective solution for reconstruction, ensuring the preservation of oral cavity function and delivering a satisfactory esthetic outcome for minor and medium-sized defects. To the best of our knowledge, there is currently no comprehensive review in the literature that simultaneously describes the use of facial vessel flaps such as FAMM, submental, and nasolabial flaps in oral cavity reconstruction. A comparison of outcomes using flaps based on facial vessels is presented in Table 2. Regardless of whether a FAMM, submental, or nasolabial flap is used, postoperative rehabilitation is essential to optimize functional outcomes.

The majority of oral cavity defects are the result of ablative surgery for oral squamous cell carcinoma (OSCC). The primary objective of oncological treatment in surgery is radical excision with adequate margin of the OSCC, often combined with neck dissection. The goal of reconstructive procedures is to achieve esthetic and functional outcomes. Nevertheless, it is essential that reconstructive surgery does not compromise the oncological radicality of excision. When using facial vessel-based flaps such as the FAMM, nasolabial, and submental flaps, oncological safety remains a frequently discussed subject. Facial vessels, due to their location on the neck, are in contact with cervical lymph nodes in levels I, II, and III. The most common site of metastasis from OSCC is the lymph nodes in level Ib, which are directly connected with the facial vein. These flaps are considered safe for use in negative-necked patients (N0). In order to ensure the success of the procedure, thorough preparation of the neck and facial vessels is essential. The issue arises when metastases are observed in the cervical lymph nodes (N+). In most cases, solitary, small metastasis without extracapsular extension (ECE) can be safely removed, and facial vessels can be preserved during therapeutic neck dissection. In contrast, multiple or large metastases with ECE have to be resected, with the sacrifice of the facial vessels. In such cases, the use of facial vessel-based flaps for reconstruction is not recommended. In this scenario, the only viable options are dpFAMM and Bozola flap with buccal artery supply [26,30]. Submental flaps are considered the least oncologically safe of the options discussed. The oncological reliability of this approach is more controversial due to the narrow diameter of the vessels, as well as the course of the submental artery and its close proximity to level I lymph nodes. These are a frequent site of early metastasis in oral cancers. In patients with clinically and radiologically N0, submental flaps can be used safely, especially in cases of posteriorly localized OSCC. However, many surgeons prefer to avoid submental flaps in oncological cases with positive or uncertain neck status, due to the potential risk of microscopic metastatic disease within the flap tissue, usually in tongue SCC [43]. 

Oral reconstruction following oncological resections is a complex field in which surgeons must carefully choose between free, regional, and local flaps, considering a range and complexity of the defect. In cases where the defect is more complex and involves bone, microvascular free tissue transfer is frequently the preferred method for reconstruction. This is due to the fact that free flap reconstructions offer enhanced versatility in the form of anatomical coverage and the ability to restore complex facial contours and improve quality of life [7,58,59,60]. However, during the coronavirus (SARS-CoV-2) pandemic, there was a shift in practice, with local pedicle flaps being used more frequently. This was due to a number of factors, including the limited availability of intensive care units and comorbidities among patients. Another issue was performing surgical procedures while wearing a special uniform that made long surgeries uncomfortable. Simpler and faster reconstruction methods were more suitable. This situation indicated practical applications in which free flap reconstructions were contraindicated [61]. Furthermore, not all defects require a free flap to achieve good outcomes, and not all patients are suitable candidates for microvascular procedures. Free flap surgeries can be associated with complications at the donor site, such as loss of the skin graft, reduced function of the donor site, and scarring. Additionally, they typically involve longer operative times, extended hospital stays, and higher healthcare costs compared to local flap procedures [45,60,62]. For medically compromised patients, facial vessel-based flaps offer a suitable alternative to microvascular reconstruction and should be considered for individuals with unfavorable characteristics such as peripheral vascular disease, bleeding diathesis, or high anesthetic risk. Therefore, local flaps provide a less resource-intensive option with lower perioperative risk.

Several pedicle flaps, in addition to those based on facial vessels, have demonstrated distinct utility in reconstructive surgery of the oral cavity. One alternative for local flap reconstruction in the oral cavity is the sublingual flap. The sublingual gland flap is suitable for small-to-medium intraoral defects in the floor of the mouth, lower gingiva, and ventral part of the tongue. It reaches the vascular supply from sublingual and submental vessels. This offers the advantage of minimal donor site morbidity. However, its limited size and mobilization restrict its use. This flap can be used as a single reconstructive option for oral defects, or to line the deeper parts of defects reconstructed using other flaps. The healing process involves granulation with unpredictable scarring, which may cause ankyloglossia [63]. Another option for reconstruction using the buccinator muscle is the Bozola flap. This flap is similar to the FAMM flap, but differs in terms of its blood supply, which comes from a buccal artery-based pedicle. The Bozola flap provides reliable vascularity, an excellent mucosal lining and good sensitivity and secretion, making it ideal for quick and easy intraoral reconstruction. It is mainly used in palate reconstruction, as well as in the posterior parts of the tongue and the floor of the mouth. One advantage of this flap is that it can be used after prior ligation of facial vessels. Secondly, modeling of the pedicle procedure can be required to achieve the best functional and esthetic results [56,64]. The buccal region can also serve as a donor site for Bichat’s flap to close intraoral defects, particularly oroantral fistulas, as well as for the reconstruction of the buccal mucosa following ablative surgery or the harvesting of large buccinator muscle flaps, such as the Bozola flap or the FAMM flap and its modifications [65]. However, this flap is mainly limited for small-to-medium-sized defects (4 cm for maxillary defects and up to 6 cm for buccal or retromolar defects), and does not provide significant volume. The Bichat fat pad flap is easy to harvest and highly adaptable, with very low morbidity and minimal failure rates. However, the healing process involves granulation, and scarring is unpredictable and may result in restricted mouth opening [65]. The palatal flap, which is based on the greater palatine artery, provides reliable mucoperiosteal tissue that matches the color and texture of the palate well. It is used to treat defects such as oronasal and oroantral fistulas, but its use is limited by the range of axial rotation and potential morbidity at the donor site, including the formation of a palatal fistula. This flap is not considered applicable for defects larger than 50% of the palatal surface [66]. Furthermore, the tongue’s rich vascular supply makes it a suitable option for repairing defects after oral cancer surgery. Common tongue flaps include the lateral and dorsal tongue flaps, based on the lingual artery and its muscular branches, and the ventral tongue flap, supplied by the sublingual artery. These flaps are particularly useful for covering exposed bone after marginal mandibulectomy and are frequently used to close oronasal and palatal fistulas. Due to their rich vascular supply, tongue flaps have a low rate of flap failure. However, they may impact speech and swallowing due to tissue division, reduced mobility, and scarring. Additionally, temporary tethering of the tongue during healing can cause discomfort and restrict oral intake [67,68].

In addition, regional pedicled flaps can be used for oral cavity reconstruction. Before the popularity of microsurgical reconstruction, large defects of the oral cavity were reconstructed using the pectoralis major myocutaneous flap. This flap remains an option for extensive reconstructions in patients with a completely depleted neck. Although the pectoralis major myocutaneous flap provides a large amount of tissue and reliable vascularity, it is associated with significant donor site morbidity and esthetic concerns. Additionally, its bulk may be excessive for smaller defects, and hair growth in the oral cavity can be an esthetically unpleasing side effect [45]. Another regional flap is the temporalis myofascial flap, which is based on the deep temporal arteries. Although this flap provides well-vascularised muscle with a long arc of rotation, making it suitable for maxillary, orbital and midfacial defects, it involves notable donor site depression and requires careful tunneling through the zygomatic arch [45]. Collectively, the examples of pedicle flaps under review demonstrate that these offer significant potential for reconstruction. It should be noted that each flap has its own particular advantages and limitations in comparison to facial vessel-based flaps, and that this allows for approaches to be customized according to the characteristics of the defect and the patient concerned.

**Table 2:** Comparison of outcomes for FAMM, nasolabial, and submental flaps in oral cavity reconstruction.

Characteristics	FAMM Flap	Nasolabial Flap	Submental Flap
**Esthetic Outcomes**	Excellent outcome in most cases No external scar No intraoral hair growth in immediate mucosal lining	Good esthetic results in most cases Extra-oral scar present Intraoral hair growth in some cases- less common after radiotherapy	Good outcome in most cases Extra-oral scar present Intraoral hair growth in some cases—hair density in the submentum and upper neck determines the esthetic results
References	[4,18]	[47,48,49,50,52]	[34,37,39,69]
**Functional Outcomes**	Normal, functional speech in most cases Regular diet possible in most cases Proper facial nerve function Risk of limited mouth opening	Normal speech function in most cases Regular diet possible in most cases Risk of buccal and marginal branch dysfunction of the facial nerve	Functional outcomes satisfactory Normal speech function and maintained swallowing Risk of marginal branch dysfunction of the facial nerve
References	[17,18,31,32]	[51,52,53,54,57,69]	[5,34,42,69,70]
**Flap Survival Rate**	Pooled success rate of 99.47% [31] 100% flap survival in 50 cases [30]	Flap survival rate of 100% [51,71] Successful reconstruction in 95% of flaps [72]	Complete flap survival in 89% [44] Complete flap survival in 93.21%, complete flap loss in 2.75% [69] 100% flap survival [43]
**Complications**	Overall complication rate, ranges around 12.8% for combined minor complications, 12.2% for partial necrosis, and 2.9% for complete necrosis [17]	Flap-related complications in 22% of patients [57]	Flap-related complications in 19% of patients [34] Possible complications ranging 0–17% caused by the intraoperative damage to the facial or margin mandibular nerve [44]
	Most commonly reported: Partial necrosis Wound dehiscence Venous congestion Rare: Complete flap necrosis Cervical hematoma Flap infection Fistula Trismus	Most commonly reported: Partial and complete flap loss Rare: Wound dehiscence Cervical hematoma Orocutaneous fistula	Most commonly reported: Partial and complete flap loss Rare: Cervical hematoma Wound dehiscence and infection Flap congestion Pharyngocutaneous fistula
References	[3,4,17,19,26]	[6,47,51,52,56,71,73]	[33,34,35,36,37,38,39,40,41,42,43,44,70]

## 5. Limitations

This review is limited by its reliance on a single database, which may have restricted the scope of available literature and excluded relevant studies indexed elsewhere. The exclusion of non-English publications may also have led to the omission of potentially important findings. In addition, the included studies demonstrated substantial heterogeneity in their reported outcomes, making direct comparison and synthesis challenging. Furthermore, many of the studies were not level I evidence, such as systematic reviews or meta-analysis, which limits the overall strength of the conclusions. Finally, the narrative review format itself carries an inherent risk of subjective interpretation and selection bias.

## 6. Conclusions

Flaps based on facial vessels, when applied with a careful and meticulously planned surgical technique, demonstrate promising outcomes in the reconstruction of oral cavity defects. The most significant advantages include a high success rate, a reduced duration of surgery and hospitalization, and a single-team approach. These flaps are a reliable option for the management of small- and medium-sized oral cavity defects, particularly in elderly patients with comorbidities.

## Data Availability

The data presented in this study are available on request from the corresponding author. Data are not publicly available due to privacy.

## Abbreviations

The following abbreviations are used in this manuscript:

FA	Facial artery
FAMM	Facial artery musculomucosal flap
iFAMM	Island facial artery musculomucosal flap
dpFAMM	Double-pedicled facial artery musculomucosal flap
